# Posterior Urethral Polyp: First Holmium-YAG Laser Ablation on a 3-Month-Old Infant

**DOI:** 10.1089/cren.2016.0037

**Published:** 2016-04-01

**Authors:** Fatih Ozkaya, Ercument Keskin, Turgut Yapanoglu, Senol Adanur, Tevfik Ziypak, Mehmet Sefa Altay, Yılmaz Aksoy

**Affiliations:** ^1^ Department of Urology, Erzurum Regional Training and Research Hospital, Erzurum, Turkey.; ^2^ Department of Urology, Mengucekgazi Training and Research Hospital, Erzincan University, Erzincan, Turkey.; ^3^ Department of Urology, Ataturk University Medical Faculty, Erzurum, Turkey.

## Abstract

***Background:*** Urethral polyps are rare benign pathologies seen in the male posterior urethra, more frequently originating from verumontanum. In this article, we aimed to discuss diagnosis and treatment of a urethral polyp causing hematuria and urinary infection in a 3-month-old male infant. This is the first case in the literature in which a urethral polyp is treated with Holmium yttrium-aluminum-garnet (YAG) laser.

***Case Presentation:*** The patient was a 3-month-old male infant, and complains were hematuria and crying during micturition. Ultrasonography and voiding cystourethrogram were used for diagnosis. Urethral polyp was observed on urethrocystoscopy. Ablation was performed with a newborn cystoscope.

***Conclusion:*** Urethral polyp can cause hematuria and urinary obstruction and should be considered in the differential diagnosis of pathologies such as posterior urethral valve and cecoureterocele that could cause infravesical obstruction. Holmium-YAG laser is a good choice of treatment with easy application possibilities using a newborn cystoscope, especially for newborns and infants who have thin urethra.

## Introduction and Purpose

Although benign, rare urethral polyps seen in the male posterior urethra cause important morbidity as they cause infravesical obstruction. Urinary infection, acute urinary retention, bilateral ureterohydronephrosis, and hematuria are some of the morbidities.^1^ Polyps are situated in the fibromuscular tissue and covered with transitional epithelium. Posterior urethral polyp causing bilateral pelvicaliceal system dilation and hematuria in a 3-month-old male infant is discussed in this article.

## Case Report

**FIG. 1.**
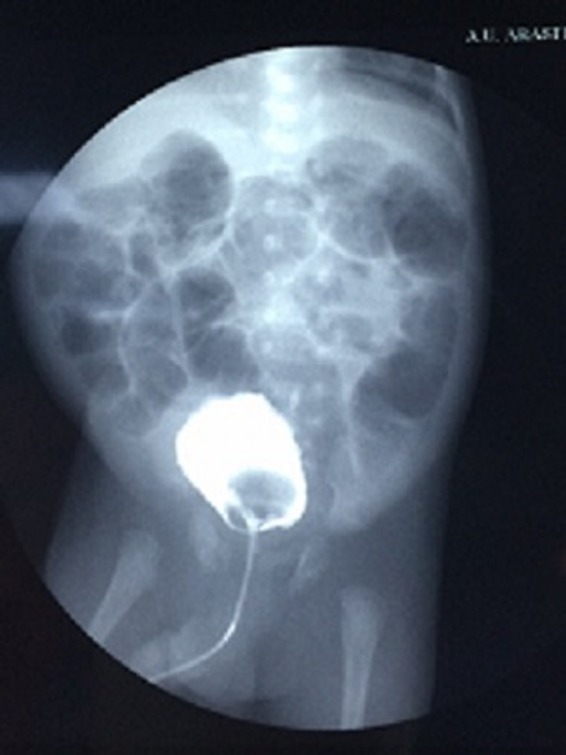
Voiding cystourethrogram.

**FIG. 2.**
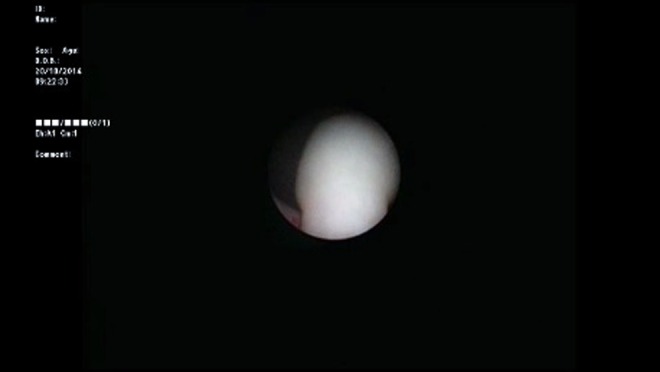
Urethrocystoscopic image.

**FIG. 3.**
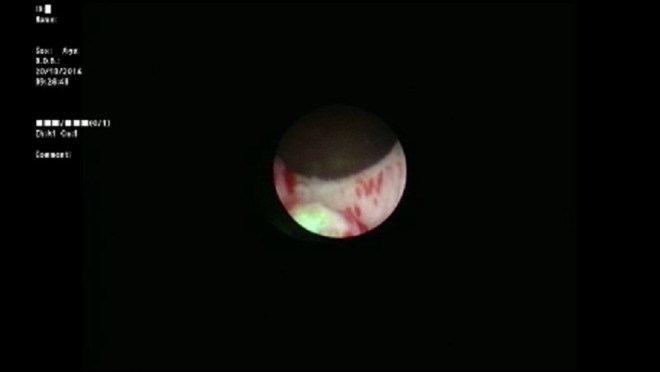
After ablation.

**FIG. 4.**
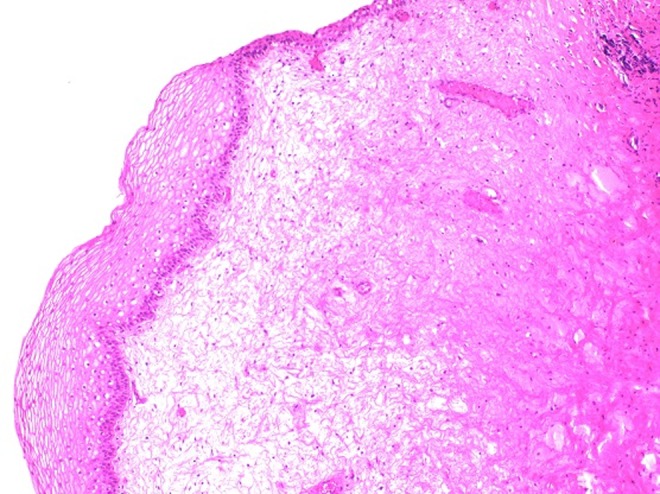
Pathology analysis “squamous metaplasia.”

A 3-month-old infant was brought to the pediatric urology clinic by his parents with complaints of hematuria and crying during micturition. It was stated that no specific findings were observed during fetal ultrasonography. Routine biochemical tests and complete blood count values were within normal ranges. No other findings besides hematuria and leukocyturia were observed in the complete urinalysis. Trabeculation in the bladder wall and a 25 × 27 mm iso-echogenous lobulized mass protruding from the bladder neck toward bladder lumen were observed on the urinary system by ultrasonography. The mean bladder wall thickness was 5.2 mm. Furthermore, mild ectasia was observed in the bilateral pelvicaliceal system. A bladder filling defect was observed on the voiding cystourethrogram (Fig. 1). Urethral polyp with dimensions of 3 × 2 cm protruding toward the bladder lumen from the posterior wall of the urethra was observed on urethrocystoscopy (Fig. 2). The polyp was ablated with Holmium yttrium-aluminum-garnet (YAG) laser using 272 μm laser probe in the same session (Fig. 3). Laser mode, power, and frequency settings were ablation, 0.8 J, 10 Hz, respectively. The polyp that was dropped into the bladder was removed as a whole piece using foreign body forceps. It was put inside formaldehyde solution and sent to the pathology laboratory. Surgical circumcision was performed because of phimosis during the same session. The transurethral drain was removed at the postoperative 8th hour and the patient was observed to spontaneously urinate with pressure. The patient was discharged. No dilation was observed in the kidneys and the bladder was normal on the control urinary system ultrasonography examination after 1 week. Pathology analysis reported as “squamous metaplasia,” unlike expected (Fig. 4). Therefore, it was decided that control urethrocystoscopy should be performed 6 months later.

## Discussion

Posterior urethral polyps are benign lesions that are rarely seen in males, are most frequently accepted to originate from verumontanum, covered with transitional epithelium, and thought to originate from mesonephric duct remnant.^1,2^ Symptoms frequently include hematuria, obstructive micturition symptoms, urinary retention, and dysuria.^3,4^ Imaging techniques, urinary system ultrasonography, voiding cystourethrogram, and urethrocystoscopy are performed in addition to routine laboratory tests for diagnosis. Transurethral resection and open cystostomy are performed generally to approach the polyps.^5^ The polyp is resected using electrocauterization or cold knife during transurethral treatment. Laser ablation has been recently begun to be used in the treatment.^6^

Neodymium: YAG laser has been used in laser ablation until now.^6^ No cases of polyp ablation with Holmium-YAG laser have been reported in the literature to date. Ablation was performed with a newborn cystoscope as the patient was too young and his urethra was thin, and as other treatment choices may have damaged the urethra. The procedure was completed with no complications as Holmium-YAG laser has a thin probe. Open surgery is performed only when the transurethral approach is not possible.^5^

The diameters of pediatric resectoscopes that are used for resection of urethral polyps are 11F–13F (Karl Storz) and 9F (Wolf). The external diameter of the newborn cystoscope that we used in our case was 6F (Wolf). A cystoscope that has a smaller external diameter than resectoscopes makes Holmium-YAG laser a good choice, which has a possibility of easy application with the newborn cystoscope on newborns and infants who have particularly thin urethra.

In conclusion, urethral polyps should be considered in the differential diagnosis of hematuria and urinary obstruction in male children. We recommend Holmium-YAG laser for treatment, as it is safe and has a good prognosis.

## Abbreviation Used

YAG: yttrium-aluminum-garnet
